# Intergenerational Transmission of Resistance of *Callosobruchus maculatus* to Essential Oil Treatment

**DOI:** 10.3390/molecules26154541

**Published:** 2021-07-27

**Authors:** Michał Krzyżowski, Bartosz Baran, Jacek Francikowski

**Affiliations:** Research Group of Insect Physiology and Ethology, Institute of Biology, Biotechnology and Environmental Protection, Faculty of Natural Sciences, University of Silesia in Katowice, 9 Bankowa Street, 40-007 Katowice, Poland; aposeris@gmail.com (B.B.); jacek.francikowski@us.edu.pl (J.F.)

**Keywords:** *Callosobruchus maculatus*, stored-product insects, resistance, insecticides, fumigant resistance, essential oils

## Abstract

Due to the rise of numerous legal restrictions as well as the increasing emergence of resistant populations, the number of available pesticides is decreasing significantly. One of the potential alternatives often described in the literature are essential oils (EOs). However, there is a lack of research addressing the potential emergence of resistance to this group of substances. In this paper, we investigated the multi-generational effects of sublethal concentrations of rosemary oil (*Rosmarinus officinalis*) on physiological and biochemical parameters of the cowpea weevil (*Callosobruchus maculatus*) such as egg laying, hatchability, oxygen consumption and acetylcholinesterase activity. Imago, which as larvae were exposed to EO at concentrations equivalent to LC_25_, showed significantly lower mortality. The results obtained indicate the potential development of resistance in insects exposed to EO in concentrations corresponding to LC_25_. In addition, in the case of the group treated with an EO concentration corresponding to LC_3.12_, a stimulation effect of the above-mentioned parameters was observed, which may indicate the occurrence of a hormesis effect. The obtained results may be an important reference for the development of future guidelines and EO-based insecticides.

## 1. Introduction

The global human population is experiencing almost continuous growth over the last decades. Most births, however, are localized in developing regions [1]. Such a state of affairs creates significant pressure on food production, storage and distribution, while even relatively low-scale destruction of crops may trigger events of extensive hunger. Such a threat, coupled with the spreading of insecticide resistance among pest species, is driving a constant demand for the development of new pesticides as well as appropriate strategies for their application [2].

One of the most promising candidate groups of substances referred to in this regard are the essential oils (EOs). Which are a broad group of volatile, plant-derived compounds commonly obtained via steam distillation of plant material [3,4,5]. The relative simplicity and cost-effectiveness [6] of EO production coupled with effective insecticidal action render them highly attractive for addressing the aforementioned demands. Notwithstanding, the research on the insecticidal usage of EOs is still an ongoing endeavor. One characteristics of EOs’ insecticidal action that has yet to be examined in detail is the question of resistance. Whether, and if so, how, EOs cause it.

*Rosmarinus officinalis* EO, which shows strong insecticidal activity against *C. maculatus*, was used based on a previous study [7]. This effect is attributed to the main constituents of the EO: 1,8-cineole (monoterpenoid), camphor (terpenoid) and α-pinene (monoterpenoid). One of the most widely accepted hypotheses for the action of *R. officinalis* EO is the ability to inhibit the AChE, which is also the main mode of action of many commercially available insecticides such as organophosphates and carbamates [8].

It is well known that during extended periods or sufficiently large scale usage of insecticides, the exposed insects’ populations undergo evolutionary pressures selecting for lower susceptibility to the used insecticidal agents. However, even in a significantly shorter period, the development of increased resistance may be observed [9], especially when initial insecticidal treatment was insufficient to successfully target all insects present in the area of application. This may lead to a scenario when the individuals (e.g., the ones undergoing larval development) exposed to sub-lethal doses of insecticide will produce the next generation.

Despite such a brief time, the successor generation of a mismanaged infestation may possess a significant degree of resistance to previously employed insecticidal agents. Thus, posing a much more formidable challenge for pest management, especially when available resources are limited.

The presented study aims to confirm whether the aforementioned effects are present in EO usage, estimating the degree of such possible influence and proposing a possible mechanism. To this end, a well-studied rosemary EO was used, and a model pest species of considerable economic significance, the cowpea weevil, *Callosobruchus maculatus*.

Obtained results may contribute to the development of effective strategies of EOs usage, counteracting the emergence of early resistance in treated *C. maculatus* populations.

## 2. Materials and Methods

### 2.1. Culture Conditions

In all assessments, mixed-sex imagoes of the *C. maculatus* were used. The insects were reared in Petri dishes on mung beans (*Vigna radiata*) in constant conditions of 30 ± 1 °C, 50% relative humidity, and the photoperiodic regime of 12/12 h light/dark.

### 2.2. Used Substance

The *Rosmarinus officinalis* essential oil used in the assessments was water-distilled and provided by local supplier Naturalne Aromaty sp. z o.o. Concentrations used in the experiments were derived from previous work on the culture [7]. On this basis, all used concentrations were calculated (LC_3.12_ = 0.1 µL, LC_6.25_ = 0.18 µL, LC_12.5_ = 0.4 µL, LC_25_ = 0.65 µL).

### 2.3. Experimental Procedure

**Figure 1:**
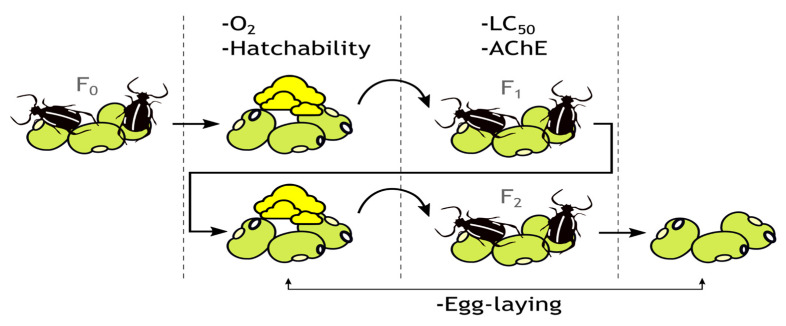
Graphical depiction of the experimental procedure.

The experimental procedure was conducted as depicted in Figure 1.

### 2.4. Fumigation

Fumigation treatment was conducted in 50 mL non-hermetic plastic containers with tight-fitting lids. For each tested concentration, ten insects (five males and five females of the same age) were placed in containers with 18 g of mung beans (four repetitions per tested group). After three days the adults were removed to prevent further egg-laying. Subsequently, the containers were incubated for five days to enable larvae to bore into the beans, in order to prevent direct exposure to EO vapors. Thereafter, filter papers (Whatman N°1) were attached to the lids of the containers and the appropriate volume of the EO was applied. For the control, no EO was used. The whole procedure was repeated for two generations.

### 2.5. Hatchability

The fumigated containers were left intact until the first adults started to emerge (22 days). Hatched insects were counted, sexed and removed from the containers every 24 h. The counting was continued until no further emergence was observed (9 days).

### 2.6. Egg Laying

The number of eggs laid for every group was assessed before each fumigation.

### 2.7. Oxygen Consumption

Five days after the start of the fumigation, infested mung beans were transferred to airtight 50 mL Falcon^®^ tubes. The oxygen consumption was measured using a SiLab data acquisition unit and oxygen sensor (sampling rate: 1/s) tightly fitted into the 50 mL Falcon tube [10]. Measurements were started immediately after putting beans into the Falcon tube and lasted for 1 h.

### 2.8. Acetylcholinesterase (AChE) Activity Assay

For each replication, 20 mg of mixed-sex insects were homogenized in Sorensen’s buffer (0.05 M; pH 7.4) in a 1:10 ratio. Thereafter, the homogenate was centrifuged (10,000 RPM, 10 min, 4 °C). Blind tests were prepared using buffers instead of homogenates. All measurements were performed with the Tecan M200 spectrophotometer in Corning^®^ 96-well UV-Transparent microplates. In the samples, the protein content was determined using the Bradford method, and then the enzyme activity was converted into Δ/min/mg of protein [11].

The AChE activity was determined by the colorimetric method of Ellman et al. [12], based on the changes in the absorbance of 412 nm light by DTNB (Ellman’s reagent) over time in the presence of AChE. The reaction mixture consisted of 150 μL DTNB (0.01 M), 20 μL AChTI (0.075 M), 10 μL probe. The eight consecutive measurements were performed every 30 s.

### 2.9. Imago LC_50_ Mortality Test

Insects that emerged after fumigation were used for the subsequent fumigation mortality assessment. Ten insects, for each repetition, were transferred to 50 mL non-hermetic, plastic containers with tight-fitting lids with filter paper attached to the lids (four repetitions for each group). The previously calculated LC_50_ concentration of EO was applied on the filter paper (Whatman N°1). Dead beetles were counted after 24 h. Insects were considered dead when no movement for 1 h was observed. For each concentration (including the control group) an additional control, consisting of untreated insects, was tested the same as other groups.

### 2.10. Statistical Analysis

The acquired data was analyzed for normality of distributions (Shapiro–Wilk test) and homogeneity of variance (Brown–Forsythe test). Based on the results of these analyses, further analyses were performed using parametric ANOVA tests. For hatching success and oxygen consumption, one-way ANOVA analyses were performed. For egg laying, hatchability, mortality and AChE activity, comparisons between groups and generations were possible, therefore, a two-way ANOVA was used. In both cases, Tukey’s multiple comparisons test was used. Analyses and graphs were prepared using GraphPad Prism v9.0 statistical software.

## 3. Results

### 3.1. Hatchability

**Figure 2:**
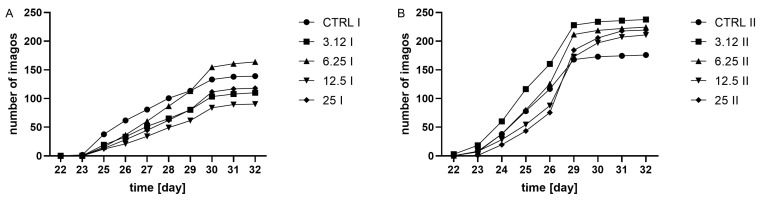
Dynamics of imago hatchability in (**A**) the first generation, I, (two-way ANOVA: F (4, 15) = 7.446, *p* = 0.0016; time F (2.038, 30.57) = 196.2, *p* < 0.0001, interaction F (36, 135) = 2.603, *p* < 0.0001) and (**B**), the second generation, II, (two-way ANOVA: treatment F (4, 15) = 3.307 *p* = 0.0394, time F (2.112, 31.69) = 564.4, *p* < 0.0001, interaction F (4, 15) = 3.307, *p* = 0.0394), cumulative plots.

Observation of adult hatching dynamics (Figure 2) revealed the time effect on variation. Moreover, in both generations, treatment was a factor differentiating groups. In the first generation, all treated groups showed a delay in adult hatching in comparison with the control. Insects from the group treated with the concentration corresponding to LC_12.5_ required the longest time to hatch. Whereas the second-generation group treated with the concentration corresponding to LC_3.12_ hatched the fastest compared to the other groups. Groups LC_12.5_ and LC_25_ required the longest time to hatch.

**Figure 3:**
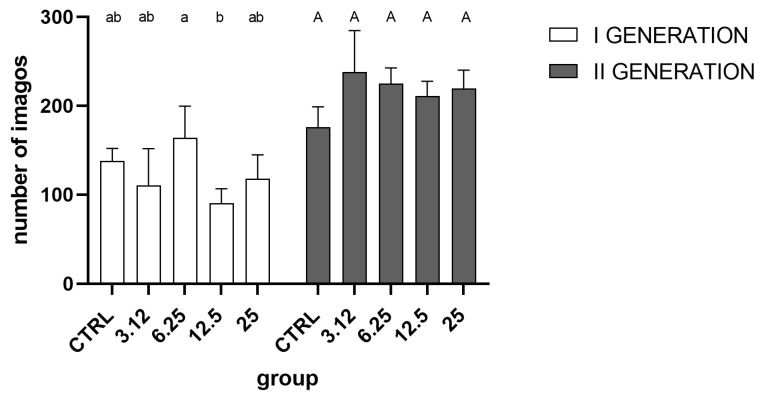
Overall hatchability of imagoes in generations I (One-way ANOVA F (4, 15) = 3.728, *p* = 0.0268) and II (One-way ANOVA F (4, 15) = 2.887, *p* = 0.0589) (mean ± SD). Tukey’s multiple comparisons test, *p* < 0.05. The letters indicate differences between groups within a generation.

Analysis of imago hatchability i.e., the total number of insects (Figure 3) indicated a weak effect of essential oil only in the first generation. The detailed intergroup analysis indicated differences only between groups treated with the concentrations corresponding to LC_6.25_ and LC_12.5_. No significant differences were observed in the second generation.

### 3.2. Egg Laying

**Figure 4:**
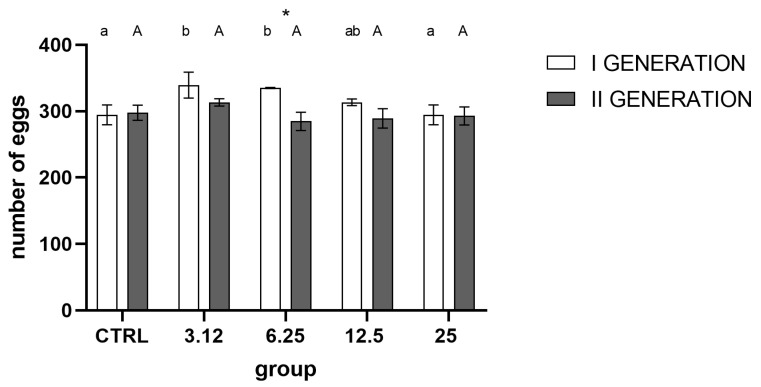
Egg laying in generations I and II, (mean ± SD) (Two-way ANOVA treatment F (4, 30) = 8.585, *p* < 0.0001, generation F (1, 30) = 24.19, *p* < 0.0001, interaction F (4, 30) = 5.685, *p* = 0.0016). Tukey’s multiple comparisons test, *p* < 0.05. The letters indicate differences within generation between groups, asterisk—differences between generations.

Analysis of the number of eggs laid by both generations (Figure 4) showed a strong effect of treatment and generation on this aspect of insects’ performance. In the first generation, there was an evident increase in reproduction in groups LC_3.12_ and LC_6.35_ compared to control and LC_25_. In the second generation, similarly, the group treated with concentration corresponding to LC_3.12_ exhibited the highest mean of all groups, but the differences were statistically insignificant. Comparison between generations within treated groups indicated higher reproduction in the first generation compared to the second generation in the LC_6.25_ treated group.

**Figure 5:**
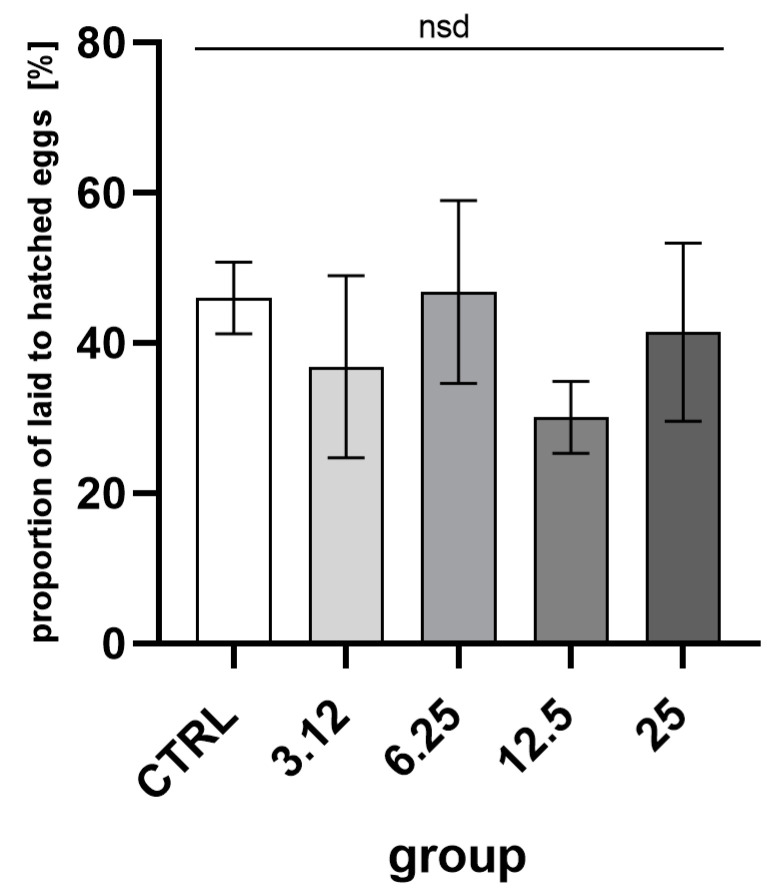
Percentage proportions of egg numbers laid by the first generation imagoes to hatched second-generation imagoes. Egg laying in the first generation in comparison with the second generation hatchability (mean ± SD) (One-way ANOVA: F (4, 10) = 1.488, *p* = 0.2774); nsd—no statistical differences between groups.

Reproductive success, the ratio of eggs laid to imagoes hatched (Figure 5), was about 40%. Comparison of groups indicated no statistically significant differences.

### 3.3. Oxygen Consumption

**Figure 6:**
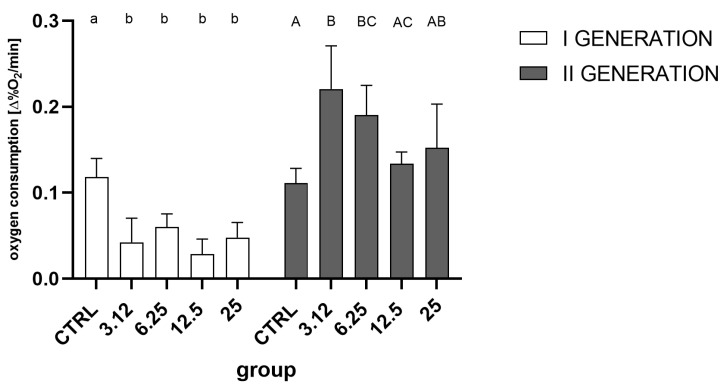
Oxygen consumption of 7-day-old larvae in the first (One-way ANOVA: 1. generation F (4, 15) = 11.47, *p* = 0.0002) and second generations (One-way ANOVA: F (4, 15) = 5.690, *p* = 0.0054). Tukey’s multiple comparisons test, *p* < 0.05. Letters indicate intra-generation differences between groups.

Analysis of the obtained data showed that in both generations, treatment with the EO had a significant effect on the change in oxygen consumption (Figure 6). In the first generation, all treated groups differed significantly from the control and were characterized by a similar reduction in oxygen consumption. However, in the second generation, contrary to the first generation, oxygen consumption in groups treated with concentrations corresponding to LC_3.12_ and LC_6.25_ was statistically significantly higher than the control, whereas groups LC_12.5_ and LC_25_ did not differ significantly from the control.

### 3.4. Imago LC_50_ Mortality Test

**Figure 7:**
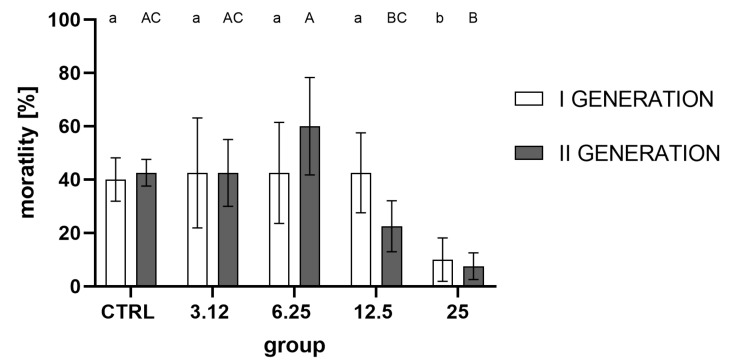
Imago mortality (%) after LC_50_ treatment corrected for negative control group mortality from the first and second generations (mean ± SD). Two-way ANOVA: treatment F (4, 30) = 11.88, *p* < 0.0001, generation F (1, 30) = 0.01408, *p* = 0.9063, interaction F (4, 30) = 2.021, *p* = 0.1167. Tukey’s multiple comparisons test, *p* < 0.05. Letters indicate intra-generation differences between groups.

Analysis of imago mortality in the acute toxicity test (Figure 7) showed a strong treatment effect on the within-group variation, while generation was not a differentiating variable. For both generations, significantly lower mortality (significantly higher resistance) was observed for insects in the group treated with the concentration corresponding to LC_25_ with respect to the control and other groups (LC_3.12_, LC_6.25_, and LC_12.5_ in the first generation).

### 3.5. Acetylcholinesterase (AChE) Activity Assay

**Figure 8:**
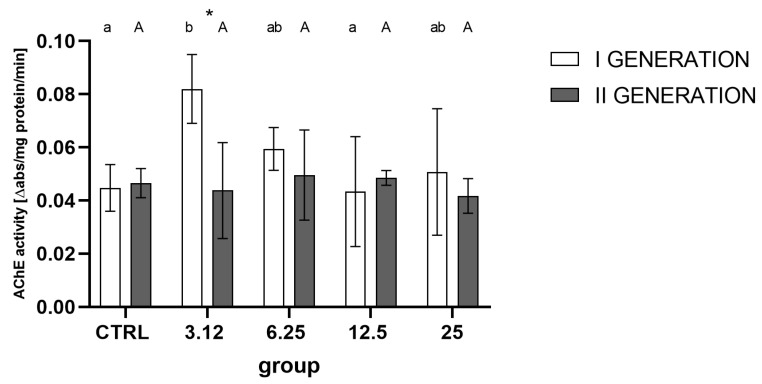
Imagoes’ AChE activity from successive generations treated with four concentrations (LC_3.12_, LC_6.25_, LC_12.5_, LC_25_) of *R. officinalis* EO (mean ± SD). Two-way ANOVA: treatment F (4, 30) = 2.301, *p* = 0.0817, generation F (1, 30) = 5.040, *p* = 0.0323, interaction F (4, 30) = 2.917, *p* = 0.0377. Tukey’s multiple comparisons test, *p* < 0.05. Letters indicate intra-generation differences between groups, asterisk—differences between generations.

Measurement of AChE activity (Figure 8) indicated a strong role of generation rather than treatment as the differentiating factor. Inter-group analysis showed a distinct increase in activity in the first generation, in the group treated with the concentration corresponding to LC_3.12_, relative to controls and the LC_12.5_ group. The second generation did not reveal significant alteration in AChE activity, i.e., there were no significant differences between groups.

## 4. Discussion

Wider (especially in the market sense) adoption of EO-based formulations in stored-products protection lags behind the growing body of research providing evidence for EOs’ effectiveness against numerous pest species [13].

Despite the aforementioned relatively extensive body of research corroborating the insecticidal effectiveness of EOs [14], there is an acute lack of studies exploring the potential adverse effects of EO usage. This, in turn, may further contribute to the aforementioned lag in adoption, as, during the development of guidelines for any pesticide usage, undesirable effects have to be accounted for. Aside from direct toxicity to non-target species or environmental danger, improper pesticide usage may also cause unwanted effects on target species. Such effects primarily include the development of resistance to applied agents. While EOs are relatively safe regarding mammalian toxicity and environmental effects, there is a significant lack of studies focusing on resistance potential in target species [15,16]. Obtaining such data is crucial for developing effective application guidelines of EO-based insecticides to avoid or mitigate the development of resistant populations.

One of the main aims of the study was to examine the impact of multigenerational exposure of insects to sublethal concentrations of EOs and the potentially resulting resistance. In this regard, one of the key aspects was the selection of the appropriate concentrations, which would be effective but yet not lethal. As the elimination of the more susceptible individuals would cause the selection of resistant individuals. Available data on the effect of EOs on egg laying and hatchability consistently indicate that exposure to those substances causes noticeable changes in those parameters [17,18]. In our experiment, used concentrations had no such effect, thus corroborating the adequate choice of proper concentrations (Figure 3 and Figure 4).

However, although the groups did not differ in the total number of hatched individuals, it was possible to observe differences in the hatching dynamics (Figure 2). This difference was observable only in the first generation, indicating the action of a stressor. In contrast, this effect was not observed in the second generation. A similar relationship was detected at the level of oxygen consumption (Figure 6), where the metabolism of larvae was significantly reduced only in the first generation. Such a pattern may indicate a metabolic load that resulted in a prolonged developmental time of the first generation.

A study by Jumbo et al. (2018) reported alterations in egg laying and hatchability of offspring after exposure of the parental generation (imago) to clove and cinnamon EOs. In the aforementioned paper, it was shown that, in contrast to males, the performance of EO-treated females decreased significantly. This may be indicative of potential egg damage, but also physiological and behavioral disturbances resulting in fewer eggs being laid. This points to the importance of selecting the appropriate developmental stage of the insect for EO treatment. Therefore, in the presented study, EO treatment was performed in the early larval stage. Which probably enabled insects to adapt to the stressor physiologically. At the larval stage, such treatment does not lead to the necrose of ova, thus allowing the oviposition of imagoes to remain unaffected.

While there were no observable differences between egg laying and hatchability, the imago mortality (Figure 7) was affected significantly in groups treated with the concentration corresponding to LC_25_ in both generations. The lack of observable effect on the levels of the egg laying and hatchability demonstrates that the observed gain of resistance could not be attributed to the selection.

It is noteworthy that there are apparent differences in response to stressors between groups treated with the highest and lowest concentrations of the used EO. A stimulating effect of the concentration corresponding to LC_3.12_ on the parameters tested (egg laying, hatchability, oxygen consumption, AChE activity) is evident. However, it is not associated with the development of resistance, as observed in the group treated with the concentration corresponding to LC_25_. One possible interpretation of such results may be the hormesis effect, in which organisms exposed to low doses of a stressor display elevated metabolic activity, survival and reproduction rates in comparison with control groups. In the case of the tested insect, only the exposure of larvae to a concentration of *R. officinalis* EO corresponding to LC_25_ constituted a sufficiently potent stressor to activate physiological resistance processes.

AChE is one of the key target enzymes in various EOs’ action [19]. Significant alteration in activity levels of this enzyme was previously observed after the treatment with *R. officinalis* EO; thus, it was assumed that its regulation might play a role in the development of a resistance response. However, the obtained data does not corroborate this hypothesis. Albeit, such an observation may also be caused by the timing of AChE activity assay—it was tested on imagoes, while the spike of AChE activity (Figure 8) may have occurred earlier, immediately after exposition to EO and subsided later, thus remaining unrecorded.

Results from the oxygen consumption assessment indicate that there is a regulation on a metabolic level. Response to the EO in the first generation was significantly reduced in comparison to the control group. Such a reduction follows the pattern observed after treatment with diatomaceous earth [20] and can occur as an initial response to the stressor. In the second generation, conversely, oxygen consumption was increased. This may lead to speculation that in the course of resistance development, at the first stage metabolic rate is lowered in order to limit the exposition and toxicity, while at the second stage, after the appropriate mechanisms are set in action, the metabolic expenditure rises in order to provide the energy for the detoxification processes.

In conclusion, the *C. maculatus* exposed to sublethal concentration of *R. officinalis* EO develops rapid resistance unrelated to selective survival of less susceptible individuals. The resistance, however, could be transferred to subsequent generations. Observed resistance most probably involves multi-stage metabolic regulation. The exact mechanisms and pathways involved in such a response remain unknown. Possible directions for further research involve testing the activity of a broader range of enzymes (especially GST and cytochrome P450 [21]), extending the experiment to more than two generations, as well as testing the possible alterations in expression and epigenetic regulation (as it may explain the observed non-selective intergenerational transfer of acquired resistance [22]) of genes involved in detoxification.

## Data Availability

Not applicable.

## Sample Availability

Samples of all used compounds are available from the authors.
